# Unintended findings: Therapeutic effects of hormones or gamma globulins on Lentiform Fork sign in 3 diabetic uremic patients: Case report/case series

**DOI:** 10.1097/MD.0000000000034819

**Published:** 2023-08-25

**Authors:** Hui Li, Wenbiao Chen

**Affiliations:** a Department of Nephrology, Shenzhen Baoxing Hospital, Shenzhen, China; b Central laboratory, People’s Hospital of Longhua, The Affiliated Hospital of Southern Medical University, Shenzhen, China.

**Keywords:** diabetic uremic patients, hormones or gamma globulins, Lentiform Fork sign, MRI neuroimaging

## Abstract

**Introduction:**

The lentiform fork sign (LFS) is a unique magnetic resonance imaging (MRI) finding characterized by a bright hyperintense rim delineating the lentiform nucleus as a fork associated with metabolic acidosis in end-stage renal disease.

**Patient concerns:**

We report 3 cases of LFS in diabetic uremic patients. In one case of uremia, intensive hemodialysis treatment was not effective. Given our poor understanding of LFS, it was regarded as bilateral basal ganglia pathology, and pulse hormone and gamma globulins therapy was initiated. The patient neurological symptoms improved, and the pathological signs on imaging subsided. Based on our experience with the first LFS case, 2 diabetic uremic cases presenting with LFS were successfully treated with hormone or gamma globulin pulse therapy in addition to intensive hemodialysis.

**Diagnosis:**

Based on the clinical manifestations, past medical history and MRI imaging changes of the 3 cases reported here, the diagnosis of LFS was established.

**Interventions:**

Our experience from these 3 cases suggests that hormone supplementation and gamma globulin therapy may be indicated for treating LFS.

**Lessons:**

Our findings highlight that in diabetic uremic dialysis patients with neurological symptoms, LFS should be suspected. The clinical manifestations, past medical history and MRI imaging findings are essential for diagnosing LFS. Hormone supplementation and gamma globulin therapy may be the effective treatment for LFS.

## 1. Introduction

The lentiform fork sign (LFS) is a unique magnetic resonance imaging (MRI) finding characterized by a bright hyperintense rim delineating the lentiform nucleus as a fork associated with metabolic acidosis in end-stage renal disease.^[1,2]^ Over the years, the LFS has rarely been documented in the literature, with only a few reports.^[3]^ Kumar et al conducted a systematic review of the imaging findings and revealed that LFS is a rare neuroimaging sign of symmetrical lesions in the basal ganglia, and the main clinical manifestations are diffuse hemispheric damage and extrapyramidal neuropathology.^[1]^ Due to the lack of understanding of LFS and systematic clinical diagnosis and treatment research, there is no effective treatment for patients with uremia combined with LFS.^[1]^ However, after symptomatic treatment, such as intensive hemodialysis and correction of acid-base and electrolyte disorders, the symptoms of most patients were relieved with normal MRI imaging results.

Here, we report 3 cases of LFS in diabetic uremic patients (Table 1). In one case of uremia, intensive hemodialysis treatment was not effective. Given our poor understanding of LFS, it was regarded as bilateral basal ganglia pathology, and pulse hormone and gamma globulins therapy was initiated. The patient neurological symptoms improved, and the pathological signs on imaging subsided. Based on our experience with the first LFS case, 2 diabetic uremic cases presenting with LFS were successfully treated with hormone or gamma globulin pulse therapy in addition to intensive hemodialysis. Our experience from these 3 cases provides novel insights for the clinical treatment of LFS in diabetic uremic patients.

**Table 1:** Clinical information of the cases.

	Case 1	Case 2	Case 3
Primary disease of renal failure	• Type 2 Diabetes	• Type 2 Diabetes	• Type 2 Diabetes
Time/yr of hemodialysis	13	3	3
Residual urine volume	0	0	0
Creatinine µmol/L	1037	368	804
Urea nitrogen mmol/L	17.45	17.16	37.44
Hemoglobin g/L	103	100	103
Parathyroid hormone pg/mL	563.0	138.6	161.7
Inorganic phosphorus mmol/L	1.86	0.94	2.6
Total carbon dioxidemmol/L	12.81	15.6	16.21
Arterial blood gas analysis	• anion gap: 27.20 mmol/L, Lactic acid concentration: 8.10 mmol/L↑。	/	/
Therapy method	Pulse hormone and gamma globulins therapy	Pulse hormone and gamma globulins therapy	Gamma globulins therapy
Clinical outcome	Improvement	Death from other diseases	Healing

## 2. Case presentation

### 2.1. Case 1

**Figure 1.**
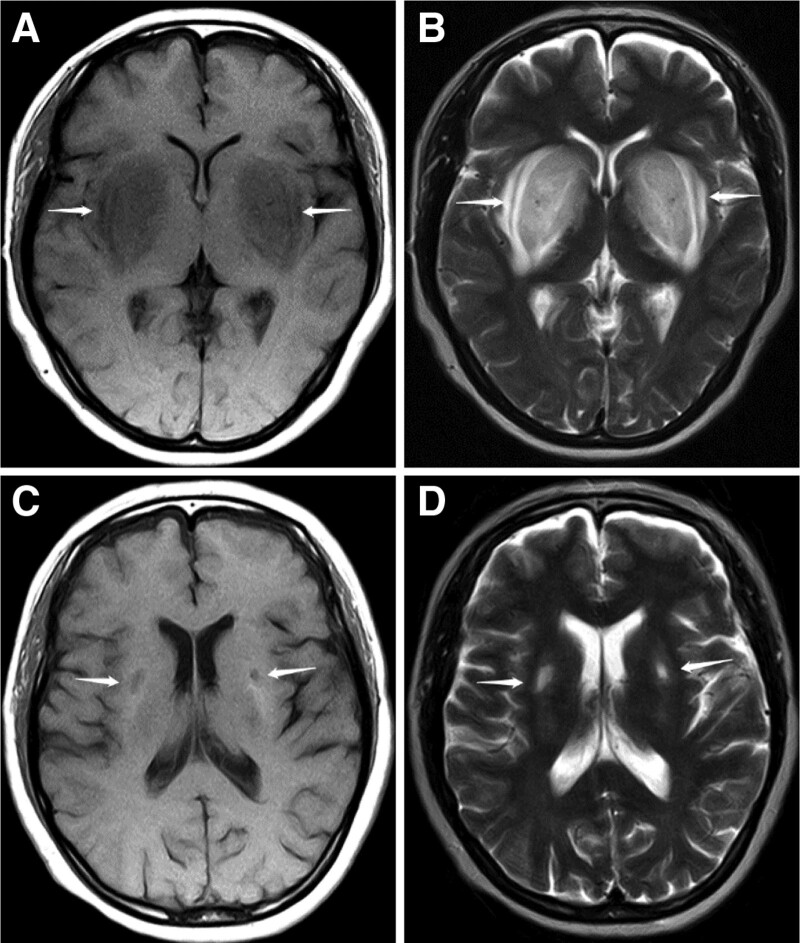
Brain MRI imaging before and after treatment in case 1. (A) T1 image of the brain shows bilateral symmetrical low-intensity signals in basal ganglia (arrow) and (B) symmetrical high signal intensity in bilateral basal ganglia on T2 images of the brain (arrow) before LFS treatment. (C) T1 images of the brain show that bilateral symmetrical signal hypo-intensities were significantly reduced in the basal ganglia with clear margins (arrow), and (D) T2 images of the brain show that the bilateral symmetrical hyperintense signals in the basal ganglia were significantly reduced, with clear margins after receiving treatment. LFS = lentiform fork sign, MRI = magnetic resonance imaging.

A 50-year-old woman who presented with slurred speech, dysgraphia, gait instability, and limb weakness for 1 week was admitted to the nephrology department of our hospital. Her past medical history was significant for end stage chronic kidney disease and underwent regular hemodialysis for 13 years and was diagnosed with type 2 diabetes 5 years ago. The patient had been taking oral repaglinide and metformin as glucose-lowering medications for nearly 1 year. Upon physical examination, the patient was conscious, with poor reactivity and slurred speech; cranial nerve examination was unremarkable, with no positive signs during chest and abdominal examination. Neurological examination revealed that the upper limb muscle strength was normal, lower limb muscle strength was decreased (grade 4), and muscle tone in the extremities was normal; deep tendon reflexes could not be elicited, and a positive Babinski sign was observed in the left extremity. Laboratory examination showed acidosis with elevated partial pressure of carbon dioxide (20.20 mmHg), plasma bicarbonate (11.9 mmol/L), actual base surplus (−11.2 mmol/L), anion gap (27.2 mmol/L), and lactic acid (8.1 mmol/L). Brain MRI showed bilateral symmetrical patchy abnormalities with blurred edges in the basal ganglia on T1 and T2-weighted MRI, which were longitudinally distributed. Hyperintense lesions were also observed on T2/FLAIR (Fig. 1A and B). Given our poor awareness of this condition, we speculated that it was associated with bilateral basal ganglia lesions, especially extrapontine myelinolysis (EPM). Therefore, in addition to intensive hemodialysis, hormone (dexamethasone, prednisone) and gamma globulins pulse therapy was prescribed and gradually tapered. After 19 days of treatment, the patient could speak fluently and normally write with clarity of thought; weakness in both lower limbs was significantly improved, and he could walk independently and smoothly. Physical examination and biochemical tests revealed no abnormalities. A repeat brain MRI results showed bilateral symmetric flaky T1 and T2 signals in the basal ganglia with clear edges that were relatively smaller (Fig. 1C and D).

### 2.2. Case 2

**Figure 2.**
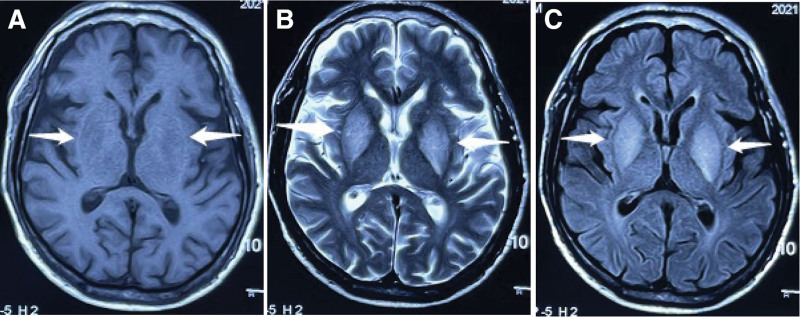
Brain MRI imaging was conducted at another hospital before hormone pulse therapy in case 2. (A) T1 MRI images of the brain show bilateral symmetrical hypo-intensity in the basal ganglia (arrow). (B) MRI T2 images of the brain show bilateral symmetrical hyperintensity in the basal ganglia (arrow); (C) FLAIR shows bilateral symmetrical hyperintensity in the basal ganglia (arrow). MRI = magnetic resonance imaging.

The second case was a 78-year-old man with diabetic uremia who received regular dialysis treatment for about a year. The patient experienced a sudden loss of consciousness and limb convulsions during hemodialysis at another hospital and was defibrillated and received cardiopulmonary resuscitation due to arrhythmia. Although sinus rhythm was restored, loss of consciousness and limb convulsions were still observed. Brain MRI showed symmetrically hypointense signals of bilateral basal ganglia on T1-weighted images, with hyperintensities on T2/FLAIR imaging (Fig. 2). After receiving intensive dialysis and symptomatic treatment at another hospital, the patient symptoms did not improve, and he was referred to our hospital for further treatment. Physical examination upon admission showed that the patient was moderately comatose with occasional eye-opening and minimal response to speech stimulus; the pupillary light reflex was negative, with a pupil diameter of 2.5mm; pression on orbit may lead to forced brain removal, limb muscle hypertonia and pathological signs could not be elicited. Biochemical studies were unremarkable. The findings of an MRI conducted at another hospital (Fig. 2) were similar to case 1 (Fig. 1). Thus, the patient was directly managed using hormone (prednisone) and gamma globulins pulse therapy and gradually tapered, along with intensive hemodialysis and symptomatic treatment. After 4 weeks of treatment, the patient was conscious and could answer, and limb twitching was significantly relieved. Unfortunately, the patient died from comorbidities.

### 2.3. Case 3

**Figure 3.**
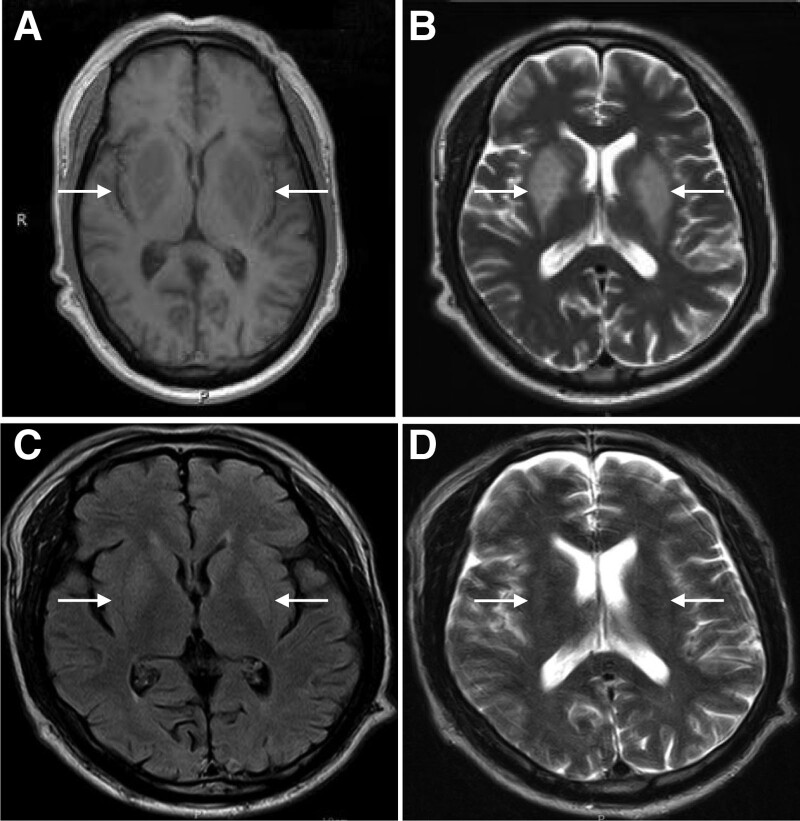
Brain MRI imaging before and after treatment in case 3. The lentiform nucleus is symmetrically swollen on both sides with long T1 and T2 signals, which indicate LFS characteristics. (A) T1 image of the brain shows symmetrical low-intensity signals in bilateral basal ganglia (arrow), while (B) symmetrical high signal intensity in bilateral basal ganglia can be seen on T2 images of the brain (arrow) before LFS treatment. No abnormal signal was found on (C) T1 (arrow) and (D) T2 (arrow) signals after gamma globulins pulse therapy. LFS = lentiform fork sign, MRI = magnetic resonance imaging.

The third patient was a 44-year-old male admitted to our hospital complaining of tremors for 1 month. The patient had a past medical history of diabetes for more than 10 years with diabetic nephropathy and underwent maintenance hemodialysis for more than 3 years. On auscultation, cardiac and abdominal examinations were unremarkable. During the neurological examination, the cranial nerve examination was unremarkable, with normal muscle strength and tone, the achilles tendon reflex could not be elicited, with bilateral symmetric hypoalgesia below the ankle joint, and pathological reflexes was negative. Laboratory examination showed metabolic acidosis with total carbon dioxide (16.21 mmol/L). Brain MRI indicated abnormal bilateral symmetrical hyperintensities of the lentiform nuclei (Fig. 3A and B). Based on our experience in cases 1 and 2, the patient was managed with gamma globulin pulse therapy, regular hemodialysis, and symptomatic treatment. After 9 days of treatment, the patient experienced no tremors, and the symptoms of limb numbness were relieved. A repeat brain MRI revealed no obvious abnormalities (Fig. 3C and D).

## 3. Discussion

LFS is a acknowledged neuroimaging finding,^[1]^ exhibiting bilateral symmetrical high signal intensities in lenticular nuclei with a fork-like shape on T2-weighted FLAIR images.^[4]^ The most frequent cause of LFS is uremia with metabolic acidosis in diabetic uremic patients,^[1]^ although the exact mechanism is largely unclear and may be related to the sensitivity of bilateral bases to toxins and metabolites.^[1]^ Indeed, it is well-established that diabetic uremic patients also present with neurovascular disease leading to damage of toxins and metabolites on neural signals and cell metabolism, eventually destroying the blood-brain barrier in the basal ganglia, leading to vasogenic edema and corresponding imaging changes.^[1,3]^ Herein, we documented 3 LFS cases. Due to our poor awareness of this disease, LFS was considered basal ganglia lesions, especially EPM.^[5]^ In addition to intensive hemodialysis and symptomatic treatment, pulse therapy with hormone and gamma globulin was prescribed, resulting in good outcomes.

Based on the clinical manifestations, past medical history and MRI imaging changes of the 3 cases reported here, the diagnosis of LFS was established. However, EPM was initially suspected based on the MRI images, given that it is well-established that EPM could involve bilateral basal ganglia lesions, with long T1 and T2 values.^[6]^ In addition, EPM is frequently associated with metabolic acidosis, especially in patients on dialysis for renal failure.^[6]^ Importantly, early treatment with high-dose of hormones or gamma globulins pulse therapy could delay the progression of EPM.^[6]^ Hormones or gamma globulins therapy was effective in these 3 cases, suggesting that LFS and EPM have similar pathophysiological changes, warranting further study. Most importantly, further emphasis should be placed on the differential diagnosis between EPM and LFS.

## 4. Conclusion

Our findings highlight that in diabetic uremic dialysis patients with neurological symptoms, LFS should be suspected. LFS should be detected and diagnosed early to avoid delaying treatment. Indeed, the clinical manifestations, past medical history and MRI imaging findings are essential for diagnosing LFS.^[1]^ Our report of these 3 cases improves our understanding of the characteristics and increases awareness on LFS. Nevertheless, the use of hormones or gamma globulins for LFS treatment may require further evidence-based medicine, emphasizing the need for more studies.

## Acknowledgments

The authors declare that they have obtained consent from each patient reported in this article for publication of the information.

## Author contributions

**Conceptualization:** Hui Li.

**Data curation:** Hui Li, Wenbiao Chen.

**Writing – original draft:** Wenbiao Chen.
